# Supplementary material for: Benchmarking Electronic-Structure Methods for the Description of Dark Transitions in Carbonyls at and Beyond the Franck–Condon Point
Source: J Phys Chem A. 2025 Sep 26;129(40):9355–67. doi: 10.1021/acs.jpca.5c05510 (PMC12516729; doi:10.1021/acs.jpca.5c05510)
Supplement: Supplementary file 1 [file jp5c05510_si_001.pdf]

# Supporting Information: Benchmarking Electronic-Structure Methods for the Description of Dark Transitions in Carbonyls at and Beyond the Franck-Condon Point

Jasmine Bone, Javier Carmona-García, Daniel Hollas,* and Basile F. E. Curchod*

*Centre for Computational Chemistry, School of Chemistry, University of Bristol, Bristol BS8 1TS, United Kingdom*

E-mail: daniel.hollas@bristol.ac.uk; basile.curchod@bristol.ac.uk

# Contents


1 Test of the basis sets S-2

2 Impact of the small basis set selected for the CC2/3 calculations S-4

3 Normalized deviations for the oscillator strengths S-5

4 Comparison of various exchange-correlation functionals along the LIIC pathway S-7

5 Active spaces for XMS-CASPT2 calculations S-8

6 Vibronically-resolved photoabsorption cross-section of acetaldehyde S-12

References S-13


# 1 Test of the basis sets

Table S1 shows the importance of diffuse functions to describe the oscillator strength for a $n\pi^*$ transition (here for acetaldehyde). Additional tests of the basis set away from the Franck-Condon point (using the LIIC described in the main text) revealed a small yet sizeable deviation for the oscillator strength obtained with aug-cc-pVDZ and aug-cc-pVTZ when reaching the region of $S_1$ (min) – see Figure S1 – motivating us to use aug-cc-pVTZ throughout this work for all electronic-structure methods tested.

**Table S1: Vertical excitation energies ($\Delta E^{el}$) and oscillator strengths ($f$) for the lowest singlet transition ($n\pi^*$) of acetaldehyde, calculated using LR-TDDFT/TDA with PBE0 and EOM-CCSD across various Dunning's basis sets.**

| | LR-TDDFT/TDA/PBE0 | | EOM-CCSD | |
|---|---|---|---|---|
| | $\Delta E^{el}$ (eV) | $f$ (x$10^{-5}$ a.u.) | $\Delta E^{el}$ (eV) | $f$ (x$10^{-5}$ a.u.) |
| cc-pVDZ | 4.34 | 0.49 | 4.43 | 1.81 |
| cc-pVTZ | 4.35 | 2.07 | 4.41 | 3.25 |
| cc-pVQZ | 4.34 | 2.68 | - | - |
| aug-cc-pVDZ | 4.30 | 3.99 | 4.37 | 5.39 |
| aug-cc-pVTZ | 4.32 | 4.47 | 4.37 | 5.54 |
| aug-cc-pVQZ | 4.31 | 4.25 | - | - |

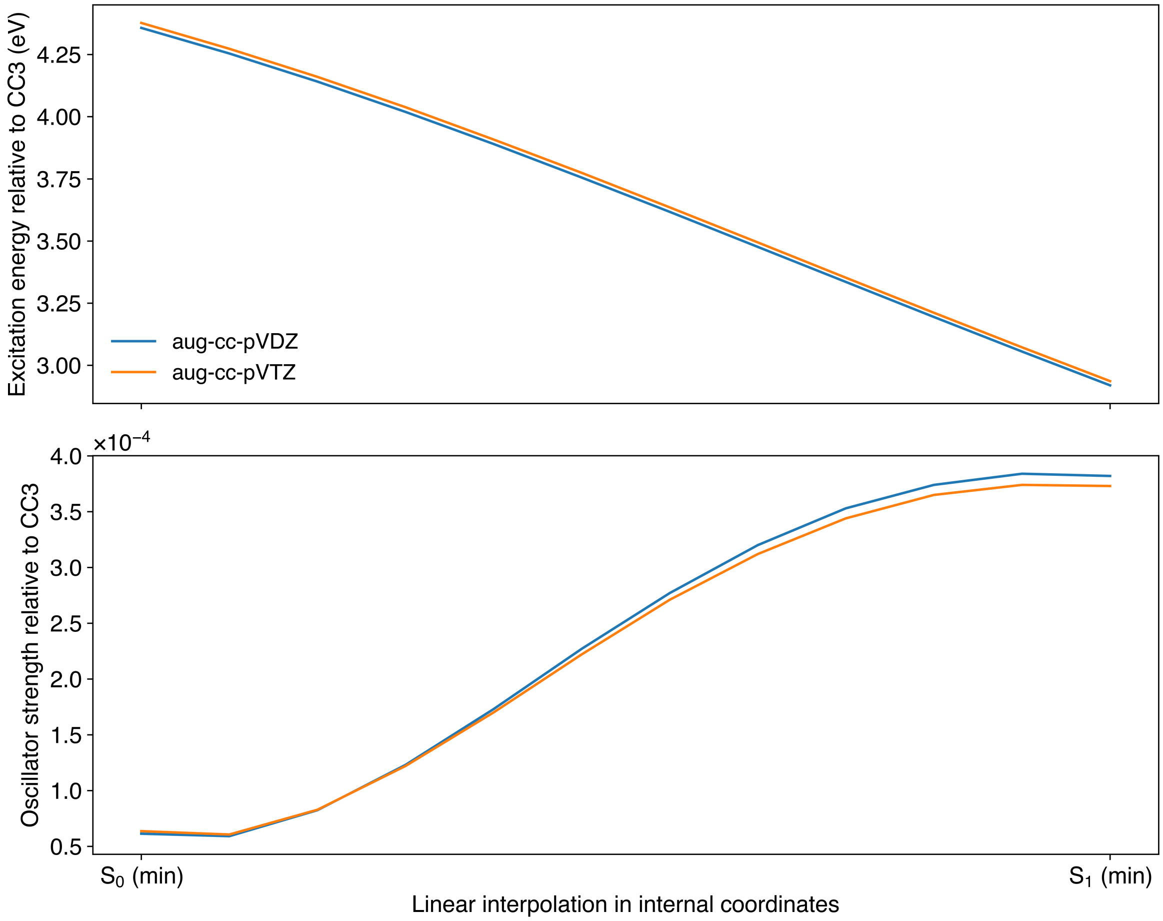


Figure S1: LIIC pathways comparing the impact of the basis set for excitation energies and oscillator strengths of acetaldehyde ($n\pi^*$ transition), calculated using LR-TDDFT/TDA/$\omega$B97X-D4 and either aug-cc-pVDZ or aug-cc-pVTZ.

# 2 Impact of the small basis set selected for the CC2/3 calculations

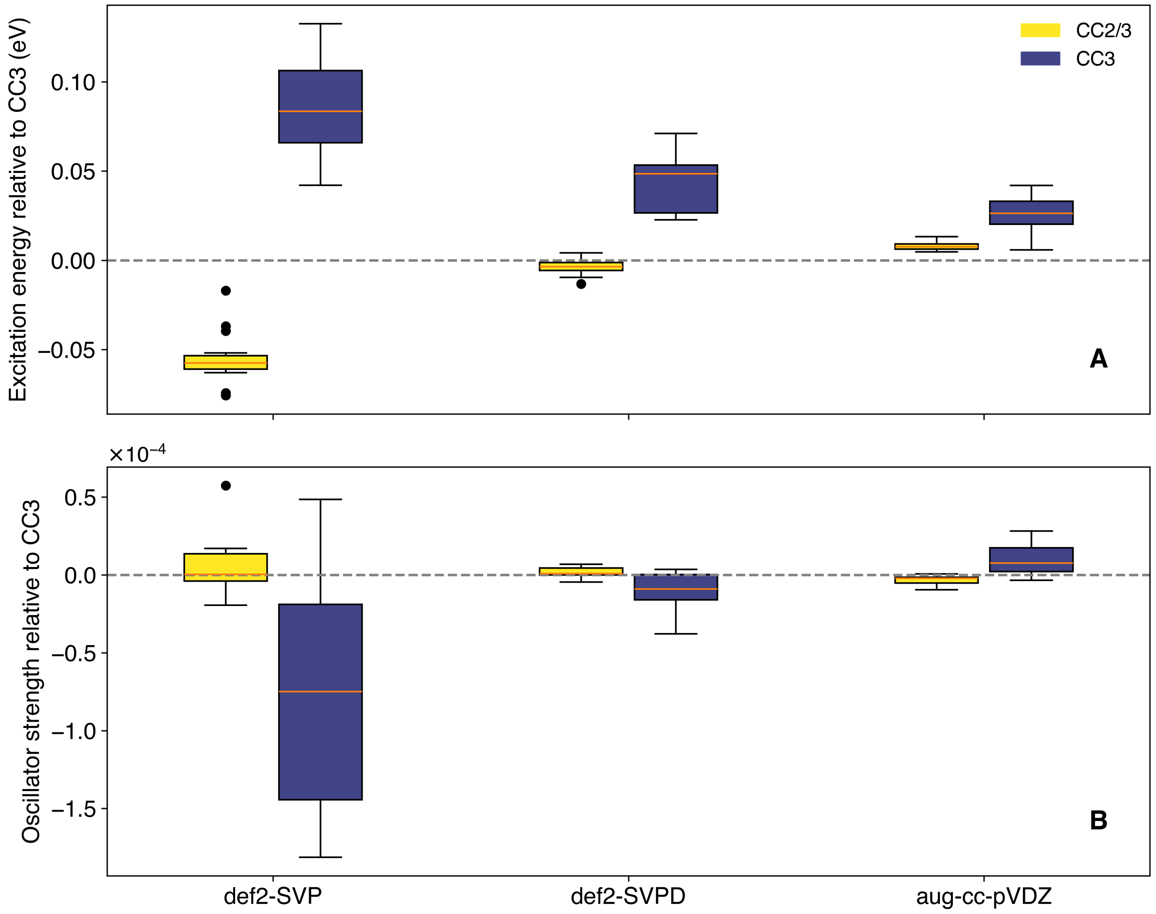


Figure S2: Interquartile range plots of the difference between CC2/3 and CC3 methods with a range of basis sets versus CC3/aug-cc-pVTZ for the excitation energies (A) and oscillator strengths (B) of the 16 carbonyl-containing molecules presented in the main text. See the caption of Figure 2 in the main text for a definition of the ranges presented. The basis sets indicated are those used for the 'low-cost' CC3 calculation, showing that diffuse functions are important to reach the full accuracy of the composite method for the $n\pi^*$ transition studied. The results for molecules exhibiting a strictly zero oscillator strength for the lowest $n\pi^*$ transition due to symmetry (formaldehyde, acetone, and cyclopropanone) were omitted from the statistics presented in panel B.

# 3 Normalized deviations for the oscillator strengths

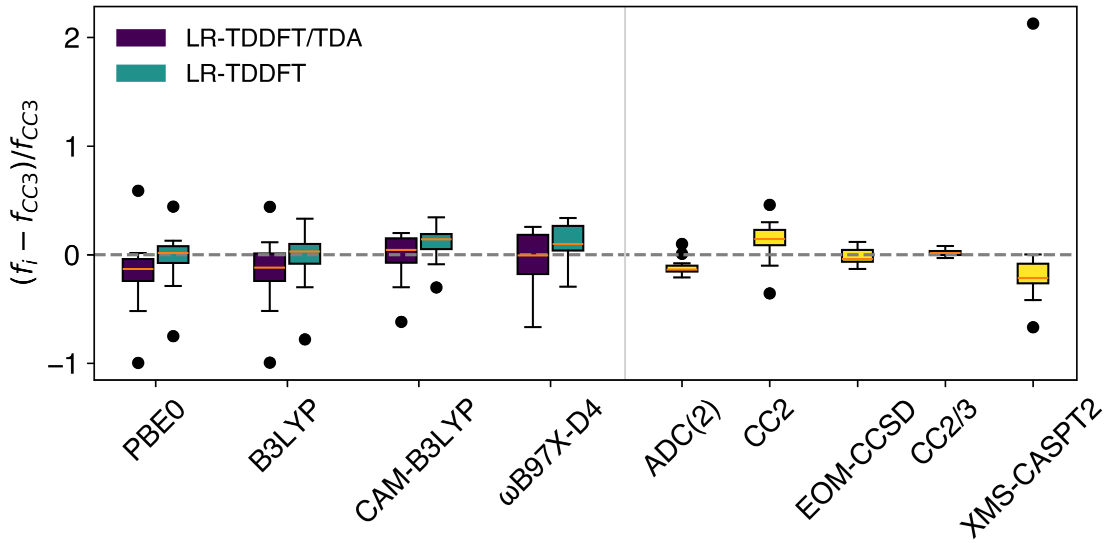


Figure S3: Interquartile range plots of the difference between a range of electronic-structure methods and CC3/aug-cc-pVTZ for the oscillator strengths of the 16 carbonyl-containing molecules (each oscillator strength was normalized by the CC3 value). See the caption of Figure 2 in the main text for a definition of the ranges presented. The results for molecules exhibiting a strictly zero oscillator strength for this transition due to symmetry (formaldehyde, acetone, and cyclopropanone) or a very large deviation (cyclobutanone) were omitted from the statistics for clarity.

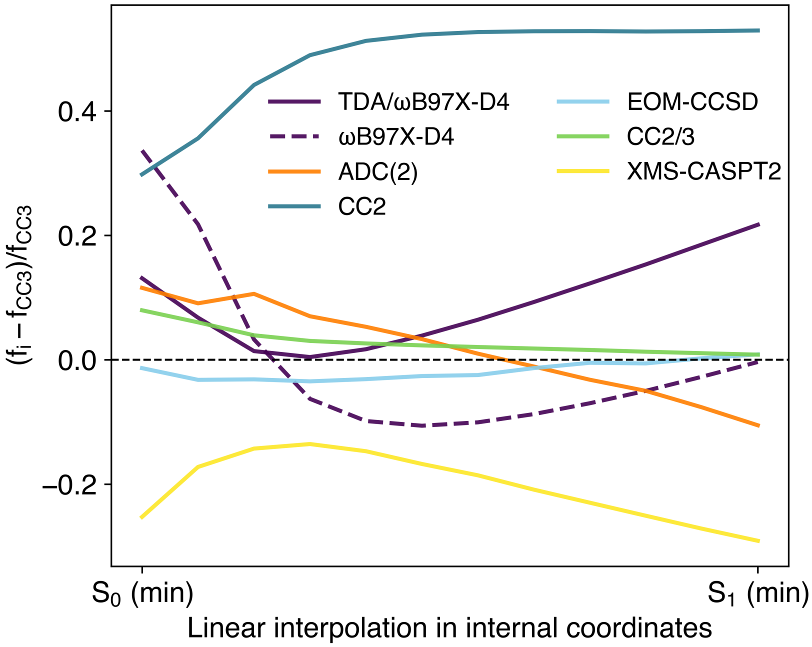


Figure S4: Oscillator strengths between $S_1$ and $S_0$ along the LIIC pathway, represented as a deviation relative to the CC3/aug-cc-pVTZ reference values normalized by the CC3/aug-cc-pVTZ oscillator strength.

## 4 Comparison of various exchange-correlation functionals along the LIIC pathway

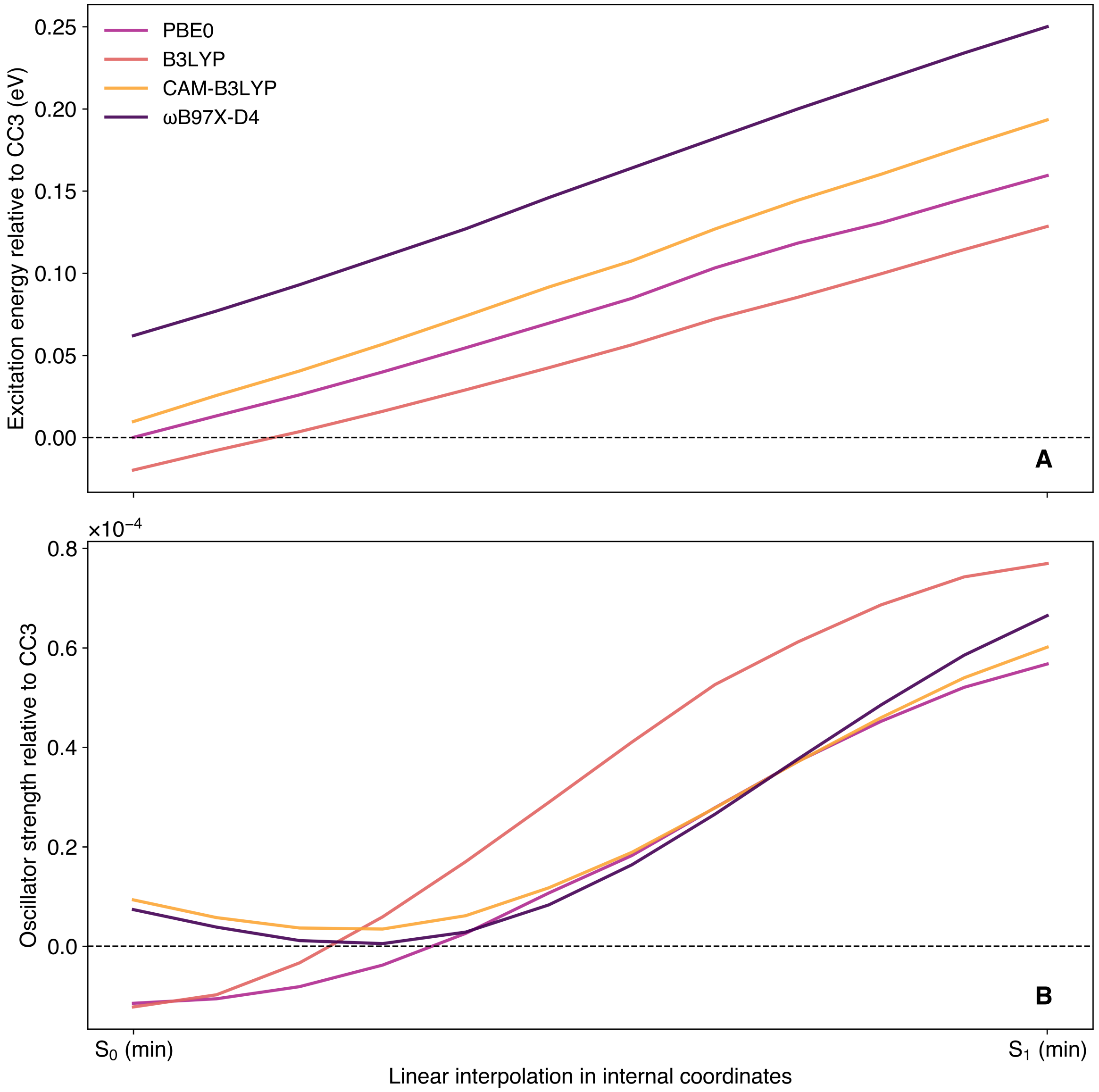


Figure S5: Test of various exchange-correlation functionals with LR-TDDFT/TDA. (A) Excitation energies between $S_1$ and $S_0$ along the acetaldehyde LIIC pathway, represented as a deviation relative to the CC3/aug-cc-pVTZ reference values (horizontal dashed line). (B) Oscillator strengths between $S_1$ and $S_0$ along the acetaldehyde LIIC pathway, represented as a deviation relative to the CC3/aug-cc-pVTZ reference values (horizontal dashed line). These calculations were conducted with ORCA v6.0.0.

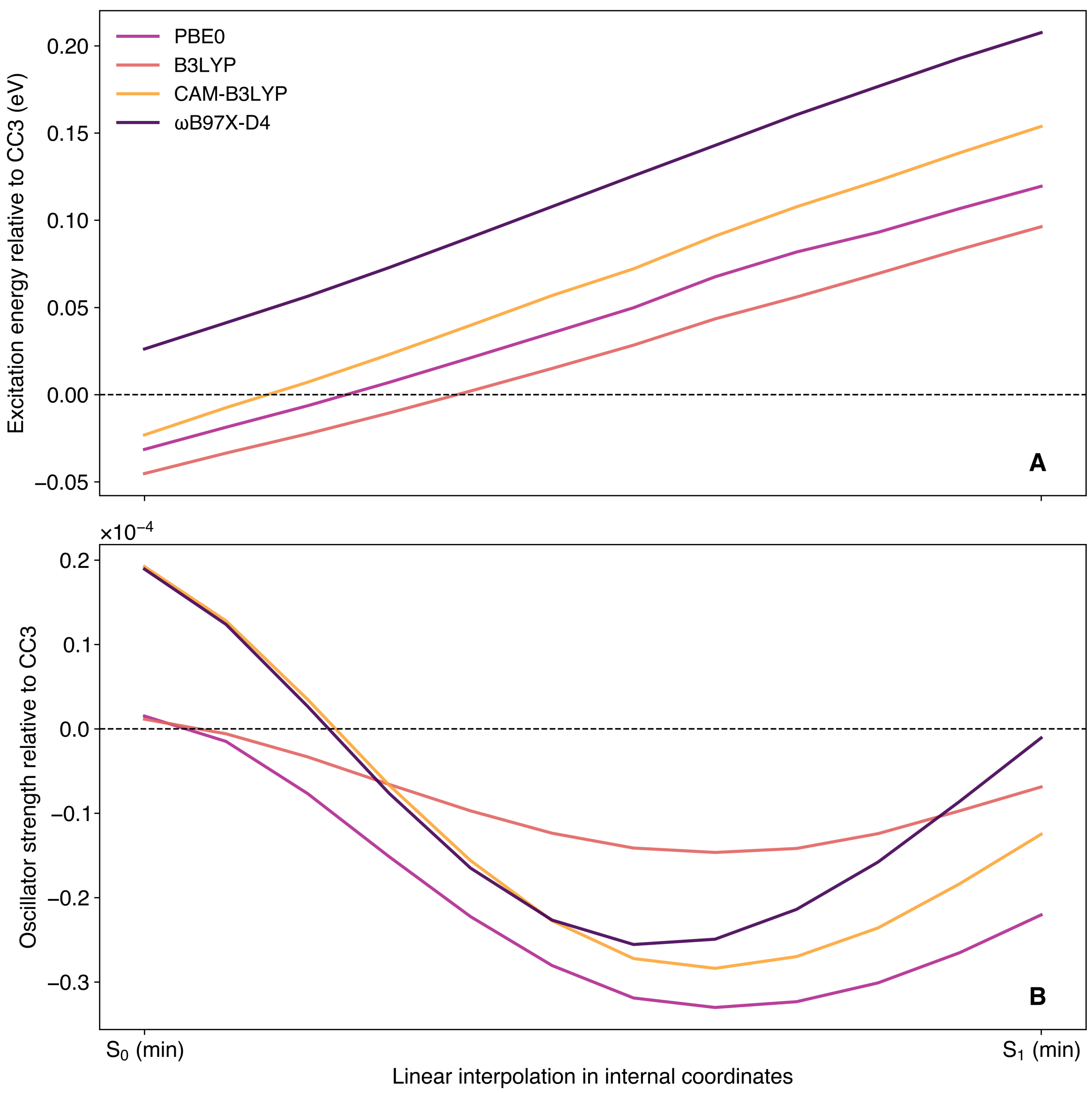


Figure S6: Same as in Figure S5, but for LR-TDDFT.

# 5 Active spaces for XMS-CASPT2 calculations

The smallest reasonable choice of active space consisted of 4 electrons in 3 orbitals ($n$, $\pi$ and $\pi^*$). This active space was employed for XMS-CASPT2 calculations on acetaldehyde, formaldehyde, acetone, trifluoroacetaldehyde, MEK, cyclobutanone, cyclopropanone, glycoaldehyde, and 3-hydroxypropanal, together with a state-averaging over two electronic states.

An example of the natural orbitals constituting this active space can be seen in Figure S7 for acetaldehyde.

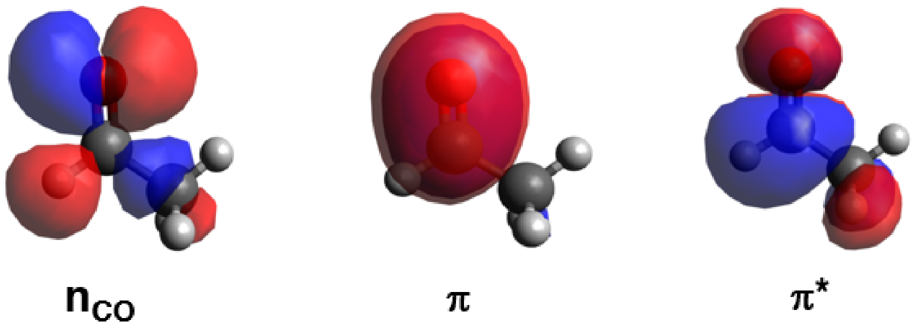


Figure S7: Active space orbitals employed in the XMS(2)-CASPT2(4/3) calculations. Natural orbitals from the SA(2)-CASSCF(4/3)/aug-cc-pVTZ reference wavefunction are given here for acetaldehyde, with an isovalue set to 0.02.

Extensions of the (4,3) active space were needed to capture the full excitation character of some molecules in the benchmark set. The (6,5) active space – used for acrolein and MVK – includes contributions from the $\pi/\pi^*$ orbitals of the alkene bond as well as the $n$, $\pi$ and $\pi^*$ orbitals of the carbonyl. An example of the natural orbitals in this active space is given in Figure S8 for MVK(I).

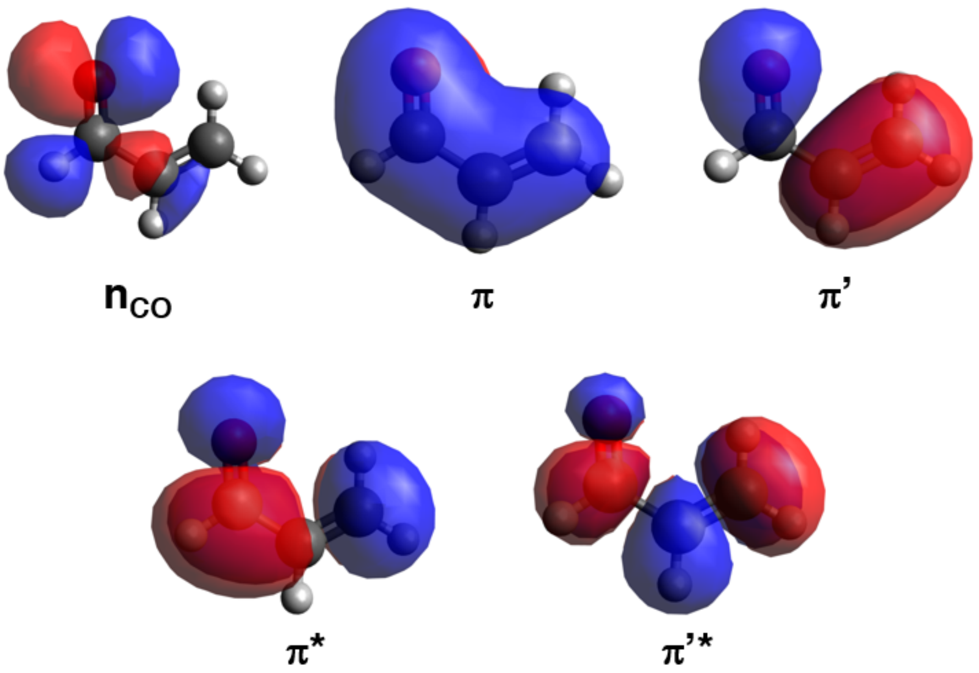


Figure S8: Active space orbitals employed in the XMS(2)-CASPT2(6/5) calculations. Natural orbitals from the SA(2)-CASSCF(6/5)/aug-cc-pVTZ reference wavefunction are given here for acetaldehyde, with an isovalue set to 0.02.

The (8,6) active space was used solely for glyoxal to capture its dialdehyde character. The active space consisted of the $n$, $\pi$ and $\pi^*$ orbitals, both in phase and out of phase, to describe adequately the key orbitals for both carbonyls. The natural orbitals included in this active space can be seen in Figure S9.

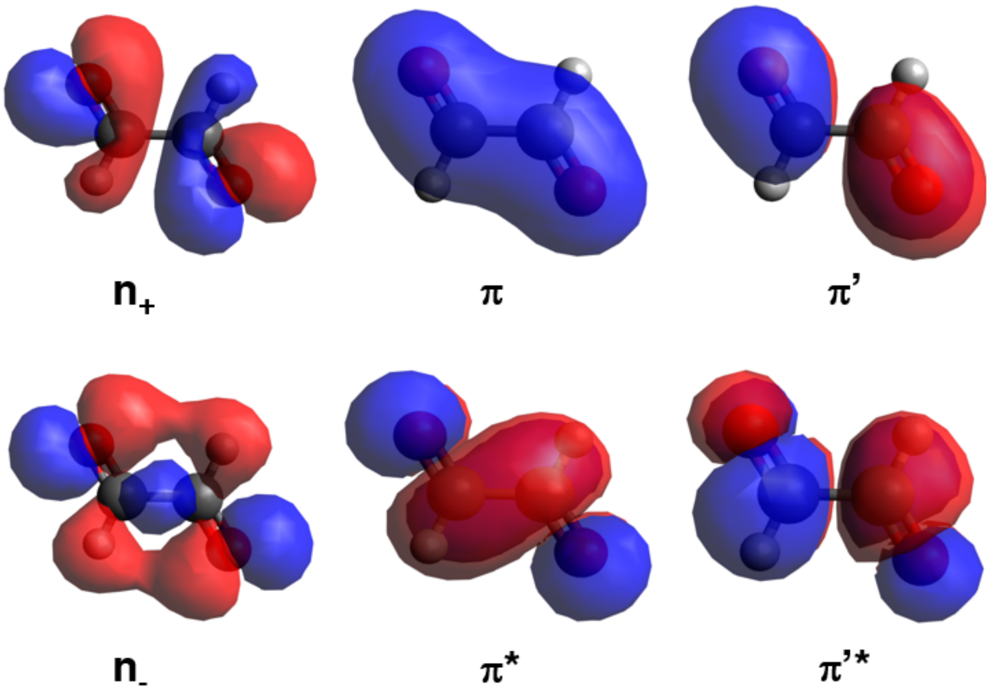


Figure S9: Active space orbitals employed in the XMS(3)-CASPT2(8/6) calculations for glyoxal. Natural orbitals from the SA(3)-CASSCF(8/6)/aug-cc-pVTZ reference wavefunction are plotted here with an isovalue set to 0.02.

For all molecules studied in this work, the $\sigma/\sigma^*$ orbitals were omitted from the active space.

# 6 Vibronically-resolved photoabsorption cross-section of acetaldehyde

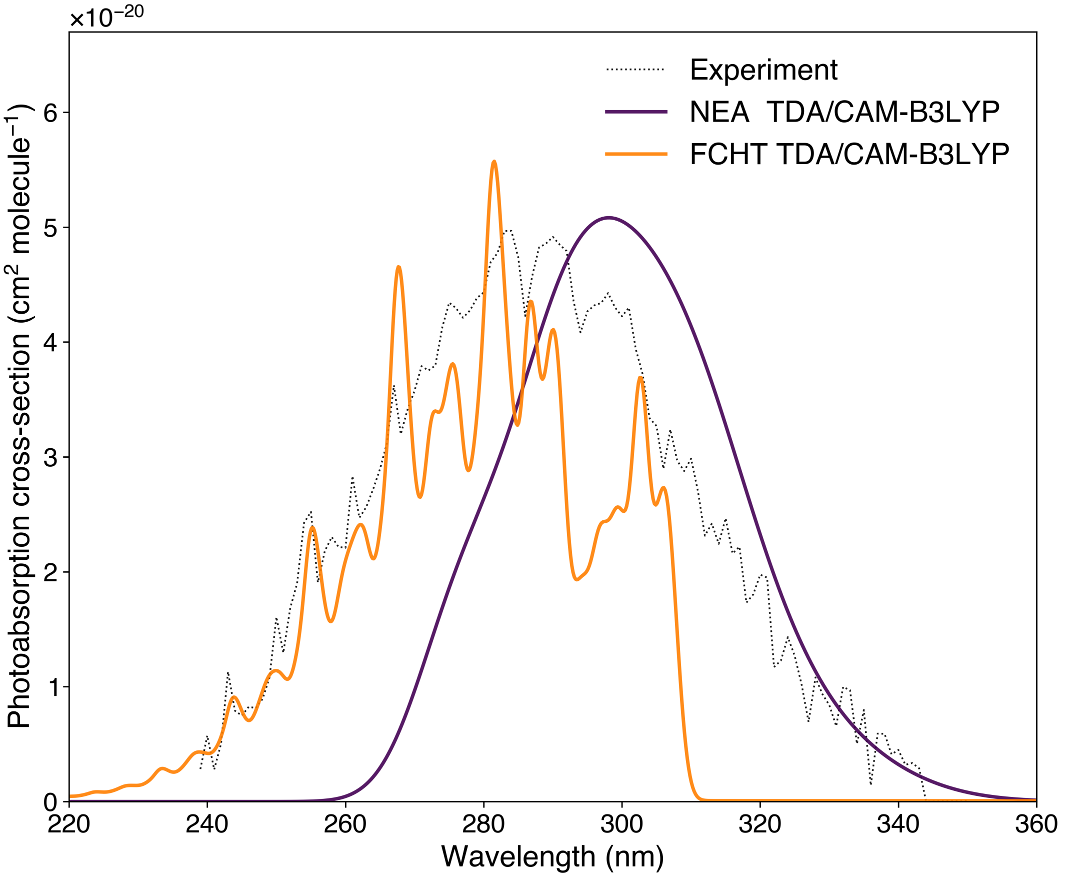


Figure S10: Photoabsorption cross-section of acetaldehyde obtained with the NEA and with the explicit calculation of Franck-Condon Herzberg-Teller (FCHT) factors. LR-TDDFT/TDA/CAM-B3LYP/aug-cc-pVTZ was used for all photoabsorption cross-sections reported. This comparison shows the role played by FCHT factors on the high-energy tail of the photoabsorption cross-section and the limited accuracy of the NEA to reproduce the full photoabsorption cross-section for bound states. The experimental photoabsorption cross-section was presented in Ref. S1 and obtained via the MPI-Mainz UV/Vis Spectral Atlas.[S2] The FCHT factors were obtained at 0 K using the vertical Hessian (and harmonic) approximation as implemented in ORCA v6.0.0, turning into real any imaginary frequency arising from calculating the Hessian of the final state at a non-stationary point, and convolving each individual transition with a Gaussian function having a half-width at half maximum (HWHM) of 0.02 eV.

# References


(S1) Limão-Vieira, P.; Eden, S.; Mason, N.; Hoffmann, S. Electronic state spectroscopy of acetaldehyde, $CH_3CHO$, by high-resolution VUV photo-absorption. *Chemical Physics Letters* **2003**, *376*, 737–747.

(S2) Keller-Rudek, H.; Moortgat, G. K.; Sander, R.; Sörensen, R. The MPI-Mainz UV/VIS spectral atlas of gaseous molecules of atmospheric interest. *Earth System Science Data* **2013**, *5*, 365–373.
